# Cross-Regional Customized Bus Route Planning Considering Staggered Commuting During the COVID-19

**DOI:** 10.1109/ACCESS.2021.3053351

**Published:** 2021-01-21

**Authors:** Ange Wang, Hongzhi Guan, Pengfei Wang, Liqun Peng, Yunqiang Xue

**Affiliations:** 1 Faculty of Urban ConstructionBeijing University of Technology 12496 Beijing 100124 China; 2 Key Laboratory of Urban Security and Disaster Engineering of Ministry of EducationBeijing University of Technology 12496 Beijing 100124 China; 3 College of Urban ConstructionHebei Normal University of Science and Technology 165079 Qinhuangdao 066004 China; 4 School of Transportation and LogisticsEast China Jiaotong University Nanchang 330013 China

**Keywords:** Customized bus, route planning, reinforcement learning, Q-learning algorithm, time window

## Abstract

In order to solve the problem of cross-regional customized bus (CB) route planning during the COVID-19, we develop a CB route planning method based on an improved Q-learning algorithm. First, we design a sub-regional route planning approach considering commuters’ time windows of pick-up stops and drop-off stops. Second, for the CB route with the optimal social total travel cost, we improve the traditional Q-learning algorithm, including state-action pair, reward function and update rule of Q value table. Then, a setup method of CB stops is designed and the path impedance function is constructed to obtain the optimal operating path between each of the two stops. Finally, we take three CB lines in Beijing as examples for numerical experiment, the theoretical and numerical results show that (i) compared with the current situation, although the actual operating cost of optimized route increases slightly, it is covered by the reduction of travel cost of passengers and the transmission risk of COVID-19 has also dropped significantly; (ii) the improved Q-learning algorithm can solve the problem of data transmission lag effectively and reduce the social total travel cost obviously.

## I. Introduction

**FIGURE 1.**
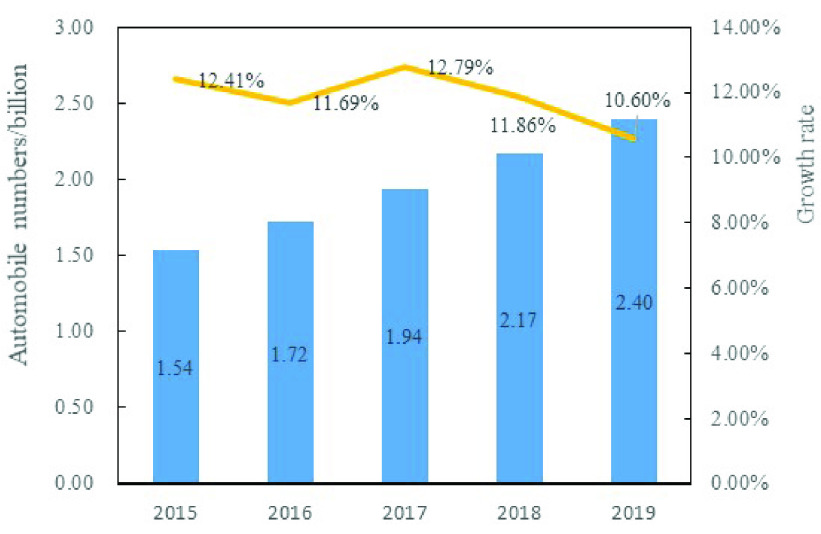
The trend of automobile numbers from 2015 to 2019 in China.

In recent years, with the rapid growth of the economy and society, the traffic congestion become more and more serious. Figure 1 is a trend chart of China’s automobile numbers from 2015 to 2019. From this figure, it can be seen from this figure that the growth rate of automobile numbers has remained above 10% from 2015 to 2019 in China. Compared with other traffic modes such as private cars and taxis, public transportation not only saves infrastructure resources, but also has irreplaceable advantages in terms of passenger transportation capacity and energy saving and emission reduction. The congestion cost of the car in Guangzhou is 1.48 *yuan/(person/km),* while the congestion cost of the bus is 0.24 *yuan/(person/km)*. For the cost of environmental pollution, the cost of the car is 2.06 *yuan/(person/km),* while the bus is only 0.004 *yuan/(person/km)* [1], so public transport travel can greatly reduce the cost of social travel. Furthermore, as people’s living standards improve, passengers have put forward higher expectations for the comfort of transportation and the convenience of transfer, the travel needs of residents have shown a trend of diversification. Besides, passengers have different travel time windows, especially during the COVID-19 period, due to the advocacy of the government and enterprises, the phenomenon of staggered commuting will become more and more common, therefore, the diversity of demand for passengers will become more and more obvious. The contradiction between the single service mode of traditional public transportation and the diversified travel needs of residents has become increasingly prominent. The development of diversified public transportation is imminent.

With the continuous construction of urbanization, the phenomenon of “separation of workplace and residence” in large cities has become more and more common. Within the six districts of Beijing, only 16.03% of the residents have achieved employment near the area where they live, 26.61% of the employees live near the area where they work. The population is mainly concentrated outside the Fourth Ring Road at 7:00 and mainly concentrated within the Fourth Ring Road at 10:00 due to work; population distribution situation at 18:00 and 23:00 are just the opposite, which means the separation of workplace and residence in Beijing is obvious [2]. The commuting time between residential and working areas is long and requires multiple transfers. Therefore, a kind of cross-regional CB that travels between residential and work areas and with low/no transfer has emerged. CB is an advanced, personalized and flexible demand response public transportation mode, which has the advantages of high reliability and comfort, as well as relative time saving [1], [2]. CB integrates passengers’ travel demands and other information, provides services for travelers with similar needs, such as starting and ending points, travel time, etc., and sets CB stops, operation routes according to passengers’ travel needs, only passengers who make a reservation in advance can enjoy the customized bus service. Therefore, the passengers’ travel demands for customized buses are relatively fixed. The ticket fares are between the regular bus and taxi prices, passengers can enjoy one seat for each person, free WIFI, no transfer and other high-quality services that cannot be provided by regular buses, which can attract more passengers and reduce the number of car trips and the traffic burden on the road.

Meanwhile, when a major public health emergency such as COVID-19 occurs, public transportation is also facing major challenges in blocking the epidemic. Cross-regional CB travel can realize direct connection between residential areas and work areas without multiple transfers, reducing the risk of disease transmission. With the normalization of epidemic prevention and control, in order to avoid excessive concentration of personnel, many companies have implemented management measures such as “staggered commuting”. Staggered commuting stipulates the commuting time of different types of employees, the purpose is to reduce the number of employees arriving and leaving at the same time, thereby reducing the risk of transmission of COVID-19. In our research, staggered commuting is reflected in the different time windows of passengers. Because we consider the travel time cost of all passengers, compared with the normal state, changes in the time window will lead to different path planning results. Therefore, how to plan a reasonable CB operation route according to the passenger’s travel time window (Due to the uncertainty of the road traffic state, it is difficult for the CB to depart/arrive at the pick-up/drop-off stops accurately at the time required by the passengers. Therefore, a small range of fluctuations in the departure/arrival time of the CB are allowed, which is called time window. If the departure time submitted by the passenger is 7:00 and the time fluctuation of 10 min is allowed, the time window is [6:50,7:10].) to reduce the total social travel cost (the summation of the bus operation cost and the passenger travel cost) is very important.

There is a growing research interest in CB route planning. In terms of theoretical research, most existing studies on CB route planning focused on the heuristic algorithm [3]–[5]. For route planning, various strategies have been proposed based on different objective functions, such as minimizing operation cost, total delay time of passengers and so on [6]–[8]. Different from the above studies, Considering the time window restrictions of pick-up stops and drop-off stops, this paper will focus on the CB route planning based on improved Q-learning algorithm which has the advantages of fast convergence speed and global optimal solution. Besides, in order to optimize the total time costs of passengers, we design a sub-regional route planning approach considering commuters’ time windows of pick-up stops and drop-off stops. Finally, we take three cross-regional CB lines in Beijing as examples for numerical experiment, the results show that the social total travel cost of the route planned by our method is greatly reduced.

**FIGURE 2.**
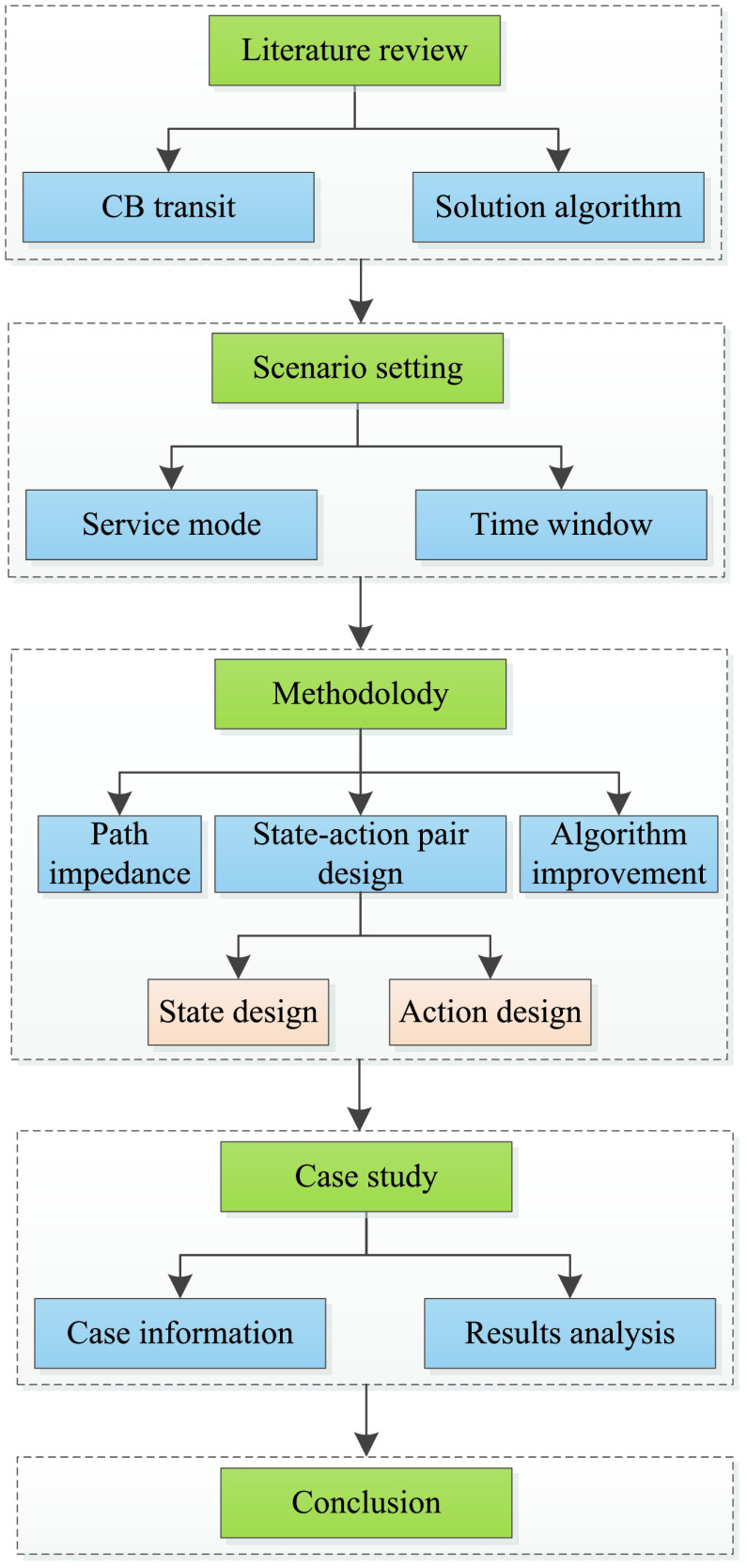
Research structure.

The remainder of this paper is organized as follows. In Section II, an overview of existing relevant research literature is summarized and the main contributions of this research is introduced. Section III describes the application scenarios in detail. Section IV introduces the improved algorithm design, and then builds the path impedance function based on historical data to obtain the optimal path between two stops. In Section V, we take three CB lines in Beijing as numerical examples. Conclusions and suggestions for future research and some proposals for CB operation are given in Section VI. The main content framework of this study is shown in Figure 2.

## II. Literature Review

This study is mainly related to the following two branches of existing literature, and contributions to this article are listed at the end of this section.

### A. CB Transit

The CB transit has been studied by some researchers. Liu and Ceder [3] presented a systematic and comprehensive analysis on a CB system. Wang *et al*. [11] constructed a multi-objective optimization model of CB routes, and clearly described the four processes of CB operation in the form of mathematical expressions, and designed a solution method based on NSGA-II algorithm. Shao *et al.* [12] established a variable-route bus dispatching two-level planning model with the goal of the largest number of people served and the smallest passenger travel time, and compared different algorithms through simulation experiments. Aiming at the shortest running time of all emergency CB lines and the constraint that passenger occupancy rate does not exceed the safety threshold, Ma *et al.* [13] built an optimization model of emergency CB routes in public health emergencies and designed a genetic algorithm to solve the model. Wang *et al*. [14] proposed three different survival models to study the mechanism of subscription behavior, among which the shared vulnerability model that considered the unobserved heterogeneity was the most appropriate. Yu *et al*. [15] introduced a method of generating bus route and stop planning suggestions based on massive demand data. A link network is generated from the input, which represents the shared path of the demand. Through community detection, the link network is divided into communities with similar travel routes. By examining the core-peripheral structure, the core part of the community is matched with the road network to generate customized bus routes. Hao *et al*. [16] constructed a complete theoretical framework that considers travel constraints and service quality satisfaction-behavior to explore parents’ choices for CB. In order to identify the origin and destination distribution of potential passengers, a spatial clustering algorithm based on pair density was proposed, using this algorithm, a method for extracting potential passengers from ordinary bus passengers based on bus smart card data was introduced [17]. Huang *et al*. [18] established a two-stage framework and optimized the bus route by establishing three nonlinear programming models. Bie *et al.* [19] proposed a mixed scheduling method combining the all-stop service and the stop-skipping service, the method optimizes scheduling strategies for multiple routes by minimizing total passenger travel time. Han *et al.* [20] presented a detailed flow chart of a CB network planning methodology, including individual reservation travel demand data processing, CB line origin destination (OD) area division considering quantity constraints of demand in areas and distance constraints based on agglomerative hierarchical clustering.

However, the above research did not consider the requirements of commuters for the time windows of the drop-off stops. In fact, commuters are more concerned about the time to arrive at the drop-off stop. In our study, we designed a route planning method for sub-regions considering the requirements of passengers’ time windows of pick-up and drop-off stops.

### B. Solution Algorithm

The algorithm for vehicle route planning can be divided into two categories: precise algorithm and heuristic algorithm. The precise algorithm includes dynamic programming method, network flow algorithm, branch and bound method, greedy algorithm, column method and so on [21]–[23]. Accurate algorithms can only solve small scale problems, and their complexity will increase exponentially with the enlargement of the problem scale, while large scale models can only be solved by heuristic algorithms. At present, the heuristic algorithms for vehicle route planning are mainly divided into two categories: population method and trajectory method. Population method mainly includes ant colony algorithm, bee colony algorithm, particle swarm algorithm, genetic algorithm and other intelligent algorithms [24]–[27], while trajectory algorithm mainly includes simulated annealing algorithm, tabu search algorithm, iterative local search, variable neighborhood search, large-scale neighborhood search algorithm and so on [28]–[30]. Since the study CB optimization problem is combinatorial that is non-deterministic polynomial-time hard (NP-hard) [31] [32]. Lyu *et al.* [8] used a variety of travel data to optimize the location, route, timetable, and the probability of passengers choosing CB. The above planning method must fit the model parameters through training data, and reflect the status of the road network through weight parameters. Randomness, according to the corresponding algorithm to solve the planned route, the training of this kind of parameter model often results in the deviation of the model parameters due to the abnormal value of the data. In the actual model, it is necessary to increase the assumptions to achieve reasonable results, and the actual problems are difficult to verify. Gao and Huang [33] used a polynomial algorithm to analyze the number of discrete path distributions and pointed out that the number of parameters has an exponential relationship with the number of road sections. Some parametric models have unsatisfactory solution results due to high parameter dimensions. In addition, some scholars have carried out research on line network optimization based on non-parametric models. Mao and Z [34] designed a non-parametric reinforcement learning model to solve the adaptive path problem in stochastic time-varying networks. Research shows that the combination of Q learning and tree-based function approximation performs better than traditional stochastic dynamic programming methods during peak demand periods. In addition, reinforcement learning algorithm is also widely used in traffic signal optimization and other fields [35]–[37]. Reinforcement learning is one of the paradigms and methodologies of machine learning, and it is a research hotspot in the field of control decision-making [38]. In the research of CB route planning, reinforcement learning is rarely combined. In practical application, Q-learning algorithm, as a classic representative of reinforcement Learning, has always been the target algorithm of great concern in various fields [39]. Nevertheless, the traditional Q-learning algorithm has the problems of slow solving speed and low efficiency [40]. Our study adopts the method of reinforcement learning to configure and optimize the CB routes, which is of great significance in improving the quality of CB service and meeting the needs of passengers for personalized travel.

### C. Objectives and Contributions

The above-mentioned representative studies have achieved rich results, but they did not consider the optimization of time windows of drop-off stops and most of them used heuristic algorithms to solve route planning problems, which have problems such as convergence to local optimal solutions and slow solution speed. Q-learning algorithm has attracted wide attention due to its low requirement for environment model and excellent self-updating ability. Besides, considering that the traditional Q-learning algorithm has the problems of slow solving speed and low efficiency, we propose a cross-regional CB route planning method based on the improved Q-learning algorithm.

The contributions of this study include: (i) we designed a route planning method for sub-regions, which can achieve the optimization of time windows of drop-off stops; (ii) the improved Q-learning algorithm where the Q value update strategy is improved to promote the algorithm efficiency is designed to solve the CB operation route with the optimal total travel cost; (iii) based on historical data, a path impedance function is constructed to determine the optimal operation path between two stops.

## III. Scenario Setting

### A. Service Mode

**FIGURE 3.**
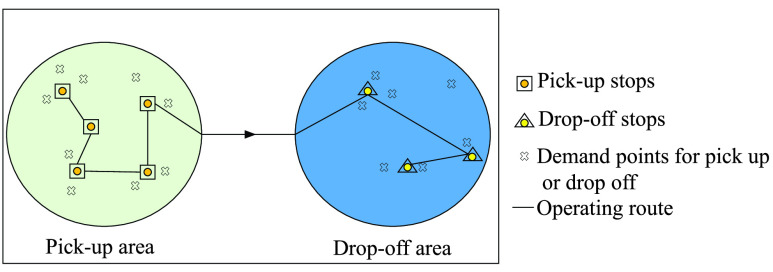
Scene graph of single region to single region service mode.

The scene diagram of this mode is shown in Figure 3, in the single region to single region service mode, we set bus stops based on travel demands at both ends of the CB line (pick-up area, drop-off area).

**FIGURE 4.**
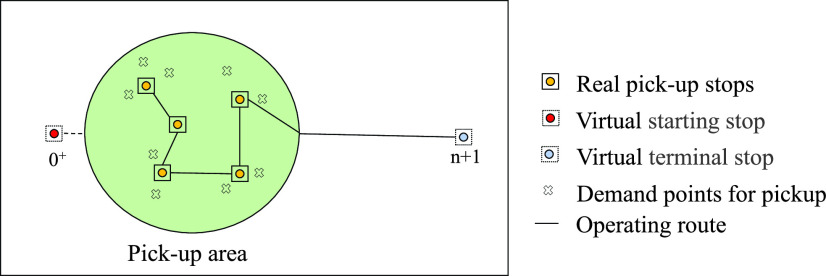
Scene graph of pick-up area.

**FIGURE 5.**
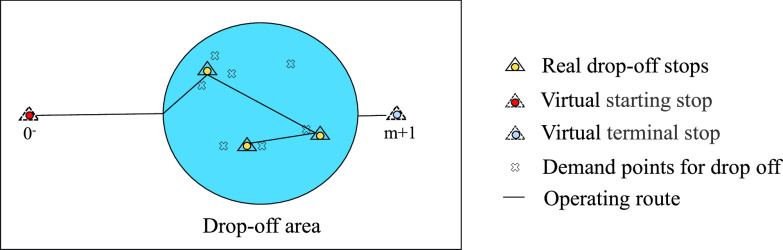
Scene graph of drop-off area.

In order to better optimize the total time costs of passengers, we transform the entire route plan into two single-region route plans. The scene graph is shown in Figure 4 and Figure 5. When solving the route of pick-up area, define $N=\left \{{{1,2,3\cdot \cdot \cdot n} }\right \}$ as the real stops in the pick-up area, and the virtual stop $0^{+}$ is set as the starting stop of the route, its distance from each actual stops is same and it is a positive number close to 0. The drop-off area is regarded as a virtual stop $n+1$ as the terminal stop of the route, and the distance between stop $n+1$ and each real stops is the actual distance. The time when the pick-up area arrives at the terminal is the departure time of the drop-off area; when solving the route of the drop-off area, define $M=\left \{{{1,2,3\cdot \cdot \cdot m} }\right \}$ as the real stops in the drop-off area, the pick-up area is regarded as the virtual stop $0^{-}$ as the starting stop of the route, the distance between it and each actual stops is the actual distance, the virtual stop $m+1$ is set as the line terminal stop, its distance from each actual stops is same and it is a positive number close to 0.

### B. Time Window

**FIGURE 6.**
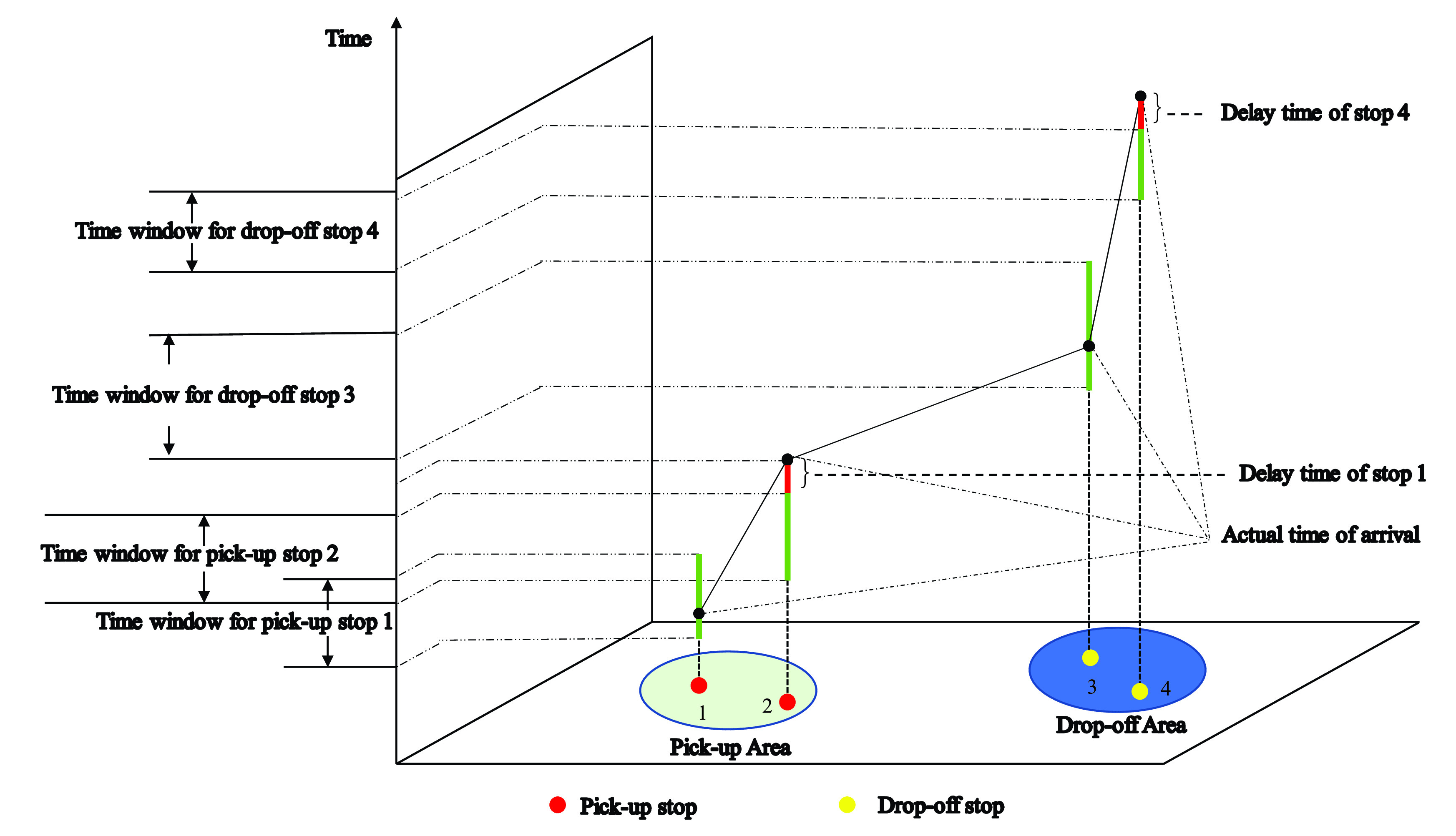
The time windows of CB’s pick-up stops and drop-off stops.

According to the actual situation, cross-regional travel for commuting has certain requirements for departure time and arrival time, so passengers will provide the platform with expected departure time and arrival time, the platform divides passengers whose departure time and location are close to the same stop, assuming that all passengers arrive at the stop on time at the specified time. The time windows of CB’s pick-up and drop-off stops are shown in Figure 6. The two-dimensional plane composed of x-axis and y-axis indicates the position of the stops, and z-axis represents time axis. If the CB arrives before or within the travel time window of the stop, there is no delay time, and the passengers have no travel time cost; otherwise, the delay time and the travel time cost of passengers occur.

## IV. Methodology

### A. Traditional Q-Learning Algorithm

The research environment of this study can be modeled as a deterministic Markov decision process, which can be viewed as a tuple $M=\left \langle{ {S,A,P,R} }\right \rangle $, where $S$ is a finite set of states, $A$ is a finite set of actions, and $P$ is the probability of moving to the next state, $R$ is the reward function, for instance, $r\left ({{s,a,s^{\prime }} }\right)$ represents the immediate reward received when agent performs action $a$ in state $s$ and move to state $s^{\prime }$.

**FIGURE 7.**
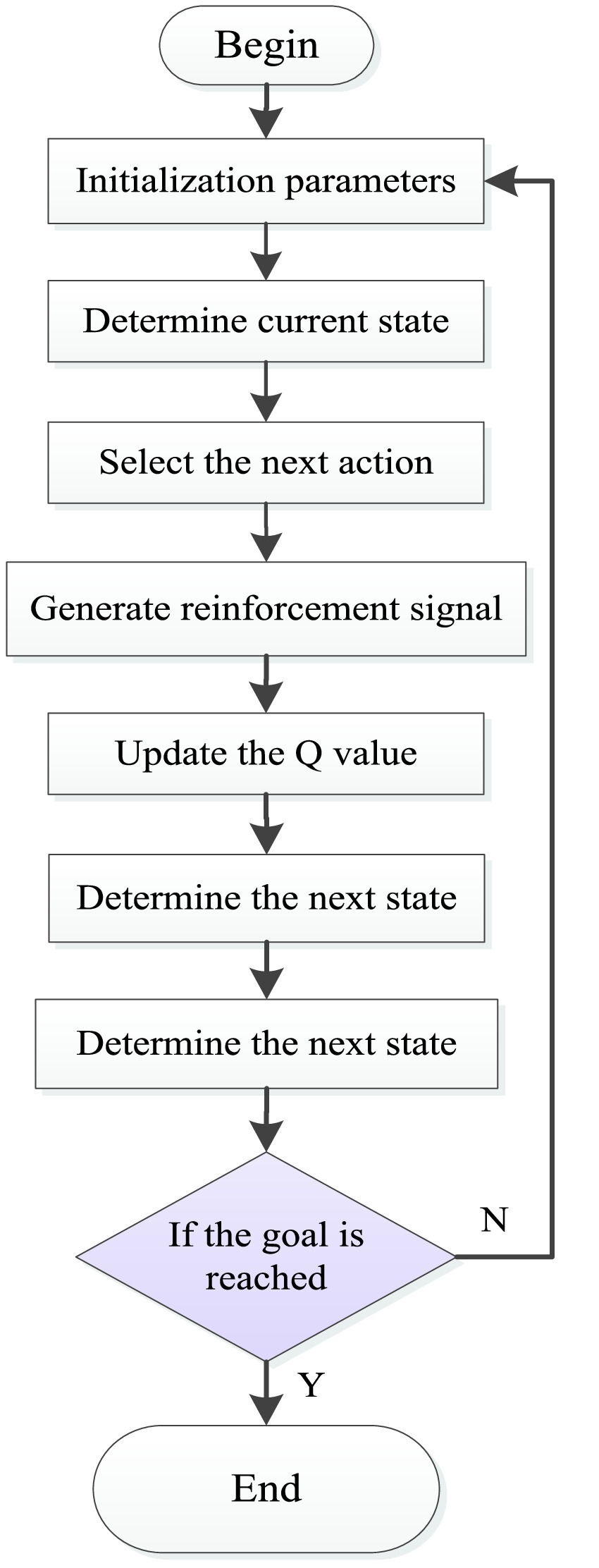
Traditional Q-learning algorithm flow.

The motivation of the learning ability of the Q-learning algorithm comes from the reward value of the environment feedback after the agent takes an action [39]. The algorithm flow of Q-learning is shown in Figure 7. The Q-learning algorithm is a value iterative algorithm. Its decision-making process and update process are related to the Q value list generated by the Q-value table. The Q-value table is a matrix of state-action pairs, when the Q values in the Q-value table do not change, it indicates that the Q-value table has converged. The establishment of the Q value list depends on the return of rewards for updating. When the agent chooses an action, the environment will transfer state and give instant rewards, correct actions will be rewarded, and wrong actions will be punished. The basic update rule is as follows:

\begin{align*} Q(s,a)=&r+\gamma \max Q(s^{\prime },a^{\prime }), \tag{1}\\ Q(s,a)\leftarrow&Q(s,a)+\alpha [r+\gamma \max Q(s^{\prime },a^{\prime })-Q(s,a)],\tag{2}\end{align*}

where $r$ is the instant reward obtained; $\gamma \in [{0,1}]$ is the discount factor which indicates the importance the agent attaches to experience, when the agent updates its state, it will comprehensively consider immediate benefits [$Q(s^{\prime },a^{\prime })$] and memory benefits [$Q(s,a)$], the memory benefits refer to the maximum value of the utility value in the action of the next state in the agent memory (Q value table). Therefore, the larger the discount factor, the more the agent attaches importance to memory benefits; $Q(s,a)$ represents the value determined by the state and action, $Q(s^{\prime },a^{\prime })$ means the Q value of the action $a^{\prime }$ taken in the next state $s^{\prime }$; $\alpha $ is the learning rate which defines how much the current q-value will move towards the direction of the latest update.

### B. State-Action Pair Design

In order to implement the Q-learning algorithm to plan the CB route, the state-action pairs and reward function of the agent learning process are the main considerations. Therefore, the first step in applying Q-learning is to define states and actions [42].

#### 1) State Design

This section is intended to illustrate: a) the CB stops are represented as states; b) the setting method of the CB stop (state).

The CB is represented as an agent, and the bus stops (including real and virtual stops) are represented as the states, the states do not change with t, because the stops are static. The real stops are set by K-Means clustering method. First, K data objects are randomly selected from the passenger travel point set. Each centroid is defined by the mean of the data objects contained in the cluster, and each remaining object is divided into corresponding clusters according to its mean distance from each cluster, the newly obtained mean point is calculated, and the clusters are re-assigned and updated until the value of each cluster. The mean value or the center of mass no longer changes. The steps of K-means cluster analysis method for cluster analysis of dynamic travel demands in a small area are:

Step 1: Process the dynamic data set and determine the location coordinates of passengers;
Step 2: Establish a reservation request data set $O$;
Step 3: Analyze the dynamic request location distribution, select $H$ initial cluster centers $Z_{h} (1,2,3...h)$ from it;
Step 4: Using $Z_{h} $ as a reference point, calculate the distance between other dynamic requests $O_{w} $ and the point $Z_{h} $, and divide the requested data into the category of the nearest cluster center, namely
  \begin{equation*} dis(O_{w},Z_{h})=\min dis(O_{w},Z_{h}).\tag{3}\end{equation*}
  If the Eq. (3) is satisfied, it means $O_{w} $ belongs to the cluster $h$;
Step 5: Update the sample points in all cluster areas $C_{h} $, and use the mean value of the sample points in all cluster areas as the new cluster center $Z_{h} $;
Step 6: According to the square error criterion, calculate the square error $E$.
  \begin{equation*} E=\sum \limits _{i=1}^{n} {\sum \limits _{p\subseteq C_{h}} {\left \|{ {p-Z_{h} ^{\prime }} }\right \|}}^{2},\tag{4}\end{equation*}
  where $p$ indicates the sample point in clustering $C_{h} $, and $Z_{h}$ is the new clustering center of clustering region $C_{h} $.
Step 7: Perform iterative calculation on the square error $E$. When the error sum of squares is locally minimum, the operation is completed; otherwise, return to Step 4.

After analyzing the dynamic travel demands using the K-means cluster analysis method, assume that the coordinate of the $w$ request in the $h$ cluster center is $(x_{rhw},y_{rhw})$, the coordinates of the composite stop are $(x_{chw},y_{chw})$, and the maximum acceptable walking distance of passengers is $d_{\max } =300m$. Determine whether the vehicle responds to the ride request according to the following formula:

\begin{equation*} \sqrt {(x_{rhw} -x_{chw})^{2}-\left ({{y_{rhw} -y_{chw}} }\right)^{2}} \le d_{\max },\tag{5}\end{equation*}

according to the service range of the ridesharing stop, the number of passengers that each cluster center accepts customized services can be determined.

#### 2) Action Design

Going to different stations means different actions. In order to avoid local optimum, the bus agent chooses different actions in each state according to the $\varepsilon \textrm {-greedy}$ strategy, that is, chooses the action with the largest Q value with probability $\varepsilon $, and chooses other actions for exploration with probability $1-\varepsilon $, and then moves to the next state. Introduce decision variables:

\begin{align*} x_{ij} =\begin{cases} 1, & {\textrm {if CB drives from station i to station j}} \\ 0, & {\textrm {other}} \end{cases}\end{align*}

when solving the routes in the pick-up area, the requirements at the end of an episode are as follows:

\begin{align*} \sum \limits _{j\in N} x_{ij}=&1, \quad {\forall i\in N}, \tag{6}\\ \sum \limits _{i\in \left \{{{0,n+1} }\right \}\cup N} x_{i,0}=&0, \tag{7}\\ \sum \limits _{j\in \left \{{{n+1} }\right \}\cup N} x_{0,j}=&1, \tag{8}\\ \sum \limits _{j\in \left \{{0 }\right \}\cup N} x_{n+1,j}=&0,\tag{9}\end{align*}

Eq. (6) indicates that the bus agent traverses all the real stops; Eq. (7) represents that the bus agent will not return to the virtual stop 0; Eq. (8) means that the virtual stop 0 is the starting stop; and Eq. (9) expresses that the virtual stop is the terminal stop.

Similarly, the conditions for the end of an episode in the drop-off area are as follows:

\begin{align*} \sum \limits _{j\in M} x_{ij}=&1, \quad {\forall i\in M},\tag{10}\\ \sum \limits _{i\in \left \{{{0,m+1} }\right \}\cup M} x_{i,0}=&0, \tag{11}\\ \sum \limits _{j\in \left \{{{m+1} }\right \}\cup M} x_{0,j}=&1, \tag{12}\\ \sum \limits _{j\in \left \{{0 }\right \}\cup M} x_{m+1,j}=&0.\tag{13}\end{align*}

### C. Path Impedance

It should be noted that the path is formed by multiple connecting road sections between two adjacent stops, route is defined as a directed line which is from the start stop to the terminal stop. However, there are often multiple alternative paths between two stops, and determining the routes between stops is the basis for constructing the public transport network. Since the paths between stops are independent and contain multiple road sections, this article uses historical data to calculate the travel time of the road section, and then determines the least impedance path.

**FIGURE 8.**
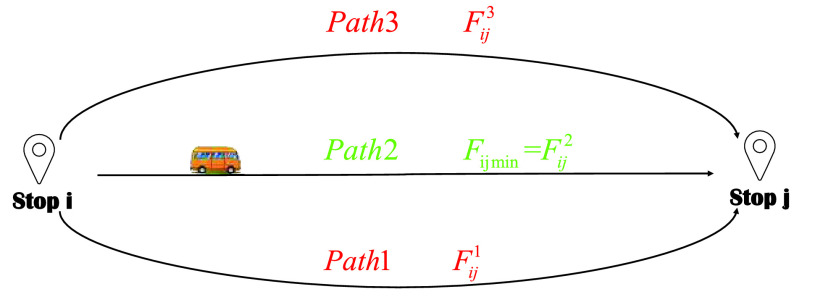
Path selection scenarios between two stops.

\begin{equation*} F_{ij} =\min \limits _{f} \sum \limits _{a\in G} {\frac {l_{a}}{v_{a}}\cdot \left [{ {1+\varphi \left ({{\frac {q_{a}}{c_{a}}} }\right)^{\varpi }} }\right]\cdot \delta _{a,f}^{ij},\quad \forall (i,j)},\tag{14}\end{equation*}

where $F_{ij} $ represents the impedance of the path $f$ between the stop $i$ and the stop $j$; $G$ means the set of road sections; $\delta _{a,f}^{ij} $ indicates the path-road section associated variable, if the road section $a$ belongs to the path $f$ between stop $i$ and stop $j$, the value is 1, otherwise the value is 0; $l_{a} $ is the length of the road section $a$; $v_{a} $ represents the free flow speed of the CB; $q_{a} $ means the actual traffic volume of the road section $a$, which can be obtained through historical data; $c_{a} $ expresses the traffic capacity of the road section $a$; $\varphi,\varpi $ are constant parameters. As is shown in Figure 8, there are three optional paths between stop $i$ and stop $j$, and path 2 has the smallest impedance, and then path 2 is selected as the operation path of stop $i$ and stop $j$.

### D. Reward Function Design

The reward function plays a guiding role in the agent training process. The purpose of this part is to evaluate the actions taken by the agent. The purpose of training is to maximize the final cumulative reward value, where the reward value is set as the opposite of the cost, that is, the higher the cost, the smaller the reward value. The reward function designed in this paper comprehensively considers the operating cost of the CB company and the travel time cost of passengers to obtain the route with the best total travel cost.

#### 1) Reward Function for Operating Costs

The operating cost of a CB company includes fixed costs and vehicle operating costs. Vehicle operating costs are positively correlated with the length of the running line. The fuel consumption cost of vehicle operation (the product of the line length and the unit fuel consumption cost) is directly used to represent its operating cost.

\begin{equation*} r_{B}^{ij} =-(l_{ij} \times p) \quad {i,j\in N}\cup \left \{{{0,n+1} }\right \},\tag{15}\end{equation*}

where $r_{B}^{ij} $ indicates the operating cost between the current stop $i$ and the stop $j$; $l_{ij} $ means the distance between the stop $i$ and the stop $j$; $p$ represents the unit fuel consumption cost.

#### 2) Reward Function of Passenger’s Time Costs

Assuming that passengers arrive at the stop at the earliest time within the time window, the time cost of the passenger is the time cost of waiting due to the late arrival of the CB (the bus arrives later than time window). Eq. (16) is the reward function of passenger’s time cost.

\begin{align*} r_{p}^{j}=&-k^{j}\left \{{{\frac {\max \left [{ {0,(t_{s}^{j} -t_{b}^{j})} }\right]}{(t_{s}^{j} -t_{b}^{j})+\ell }\times (t_{s}^{j} -t_{b}^{j})\times u_{p}} }\right \} \tag{16}\\ t_{s}^{j}=&t_{s}^{i} +F_{ij},\tag{17}\end{align*}

where $r_{p}^{j} $ represents the time cost of passengers at the stop $j$; $k^{j}$ is the number of passengers at the stop $j$; $t_{s}^{i} $ means the actual time for the CB to arrive at the stop $i$; $t_{s}^{j} $ represents the actual time for the CB to arrive at the stop $j$, that is, the sum of the time of arrival at the stop $j$ and the path impedance (operation time) between the stop $i$ and the stop $j$; $t_{b}^{j} $ indicates the latest time in the time window of stop $j$; $u_{p} $ means the time value of passengers. According to the actual situation, the time value of passengers in the drop-off area is higher than that in the pick-up area; $\ell $ is a positive number close to 0, avoiding the denominator being 0.

If the actual arrival time of CB is within the time window of the stop, $(t_{s}^{j} -t_{b}^{j})$ is negative, therefore $\max \left [{ {0,(t_{s}^{j} -t_{b}^{j})} }\right]$ is 0, there is no delay time cost; If the actual arrival time of CB is after the latest time in the time window of the stop, $(t_{s}^{j} -t_{b}^{j})$ is positive, therefore, $\max \left [{ {0,(t_{s}^{j} -t_{b}^{j})} }\right]$ is $(t_{s}^{j} -t_{b}^{j})$, $\frac {\max \left [{ {0,(t_{s}^{j} -t_{b}^{j})} }\right]}{(t_{s}^{j} -t_{b}^{j})+\ell }$ is approximately equal to 1, the delay time occurs. Therefore $\frac {\max \left [{ {0,(t_{s}^{j} -t_{b}^{j})} }\right]}{(t_{s}^{j} -t_{b}^{j})+\ell }$ is essentially a “decision variable” to judge if the CB arrives stop outside the time window, then the reward value of passenger’s time cost for the bus agent is determined by Eq. (16). In both the pick-up area and the drop-off area, passengers require that the actual arrival time of CB should not be later than the time window of the stop, $(t_{s}^{j} -t_{b}^{j})$ means the difference between the actual arrival time and the latest time in the time window. Hence, Eq. (16) can describe the time deviation between the pick-up area and the drop-off area.

#### 3) Comprehensive Reward Function

The optimization goals of Eq. (15) and Eq. (16) have the same direction, and the multi-objective optimization is transformed into single-objective optimization, and the final reward function is:

\begin{align*}&\hspace {-.5pc} r=-\omega _{1} \left \langle{ {k^{j}\left \{{{\frac {\max \left [{ {0,(t_{s}^{j} -t_{b}^{j})} }\right]}{(t_{s}^{j} -t_{b}^{j})+\ell }\times (t_{s}^{j} -t_{b}^{j})\times u_{p}} }\right \}} }\right \rangle \\&-\,\omega _{2} \left ({{l_{ij} \times p} }\right),\tag{18}\end{align*}

where $r$ indicates the reward function; $\omega _{1},\omega _{2} $ respectively are the weight coefficients of operating cost and passenger time cost [20], the other variables and parameters have been explained above.

### E. Algorithm Improvement

The Q-learning algorithm has the problem of data transmission lag in the Q value update process, which causes the Q value of the subsequent state to not be fed back to the forward state in time to update the Q value, which affects the convergence speed of the Q value. In this study, the “backtracking” idea is used to improve the update rules of the Q-value table to solve the problem of data transmission lag, improve the convergence speed of the algorithm, and shorten the path planning time.

First define the memory matrix $M\left ({t }\right)\leftarrow \left [{ {s_{t},a_{t}} }\right]$ to record the state-action pairs experienced by the bus agent in sequence. Suppose $M\left ({t }\right)\leftarrow \left [{ {s_{t},a_{t}} }\right]$ is a matrix with rows and 2 columns, at the beginning of training, since no prior experience is set for the agent, so the initial input of all Q values in the memory matrix is 0. where $z$ is the number of states experienced from the initial moment to the current moment. Use $\left [{ {s_{t},a_{t}} }\right]$ in $M\left ({t }\right)\leftarrow \left [{ {s_{t},a_{t}} }\right]$ as the index to find the Q value corresponding to the previous “state-action” and update it. Then subtract 1 from $t$ and determine whether $t-1$ is 0. If it is 0, it means that the Q value of all the “state-actions” experienced by state $s_{t} $ has been updated; if it is not 0, continue to index the previous sequence “The Q value of “state-action” is updated until all Q values are updated, so that the Q values of n states are updated without repeated training n times. The update formula is shown in equation (19), where $k=t-1,t-2,\ldots,2,1$.

\begin{align*}&\hspace {-.5pc} Q\left ({{s_{k},a_{k}} }\right)\leftarrow Q\left ({{s_{k},a_{k}} }\right) \\&+\,\alpha \left [{ R_{k}+ \gamma \max \limits _{a} Q\left ({{s_{k+1},a} }\right)-Q\left ({{s_{k},a_{k}} }\right) }\right],\tag{19}\end{align*}

### F. Algorithm Flow

**FIGURE 9.**
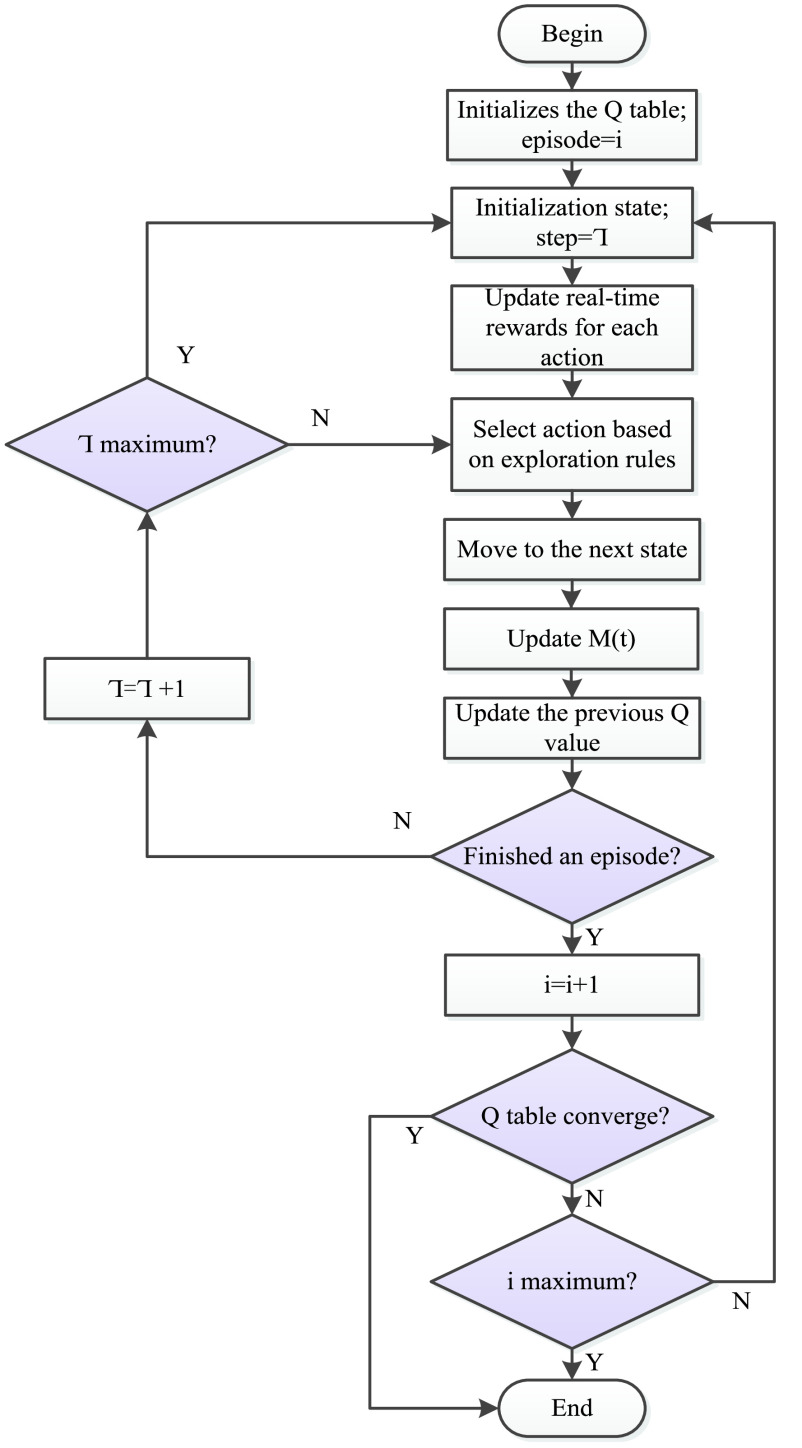
Improved Q-learning algorithm flow.

Since the reward and punishment value includes the passenger’s time cost, which is related to the actual time when the bus arrives at the stop, the reward and punishment value of each action in this state must be recalculated every time it reaches a state. A step-by-step procedure to search for the optimal route using the improved Q-learning algorithm is shown in Figure 9 and discussed below.

Step I: The initial input of all Q values in the Q value table is 0, and let ${{\Gamma }} = 1$ to start the first training.
Step II: Initialize the state, and let ${{\Gamma }} =1$.
Step III: Update the reward value for all actions based on the current state.
Step IV: Select the next action according to $\varepsilon \textrm {-greedy}$ strategy, then move to the new state.
Step V: Update the memory matrix $M\left ({t }\right)$ and previous Q value.
Step VI: Determine if an episode has been completed according to the set training conditions. If an episode is not completed, let ${ {\Gamma }} = { {\Gamma }} +1$, and then judge if the maximum number of training steps has been reached, if so, go back to Step II; otherwise go back to **Step IV**. If an episode is completed, let i = i +1 and then go to **Step VII**.
Step VII: Determine if the Q-value table has converged (the Q values in the Q-value table do not change). If Q-value table does not converge, it is judged if the maximum episode number has been reached, if the maximum number of episodes is reached, the training ends, if the maximum number of episodes is not reached, go back to **Step II**. If Q-value table has converged, the training ends.

## V. Case Study

### A. Line Information

We take three existing customized bus lines in Beijing as examples to verify the effectiveness of the proposed algorithm. These three CB lines are typical cross-regional CB lines, and they are similar to other cross-regional CB lines in Beijing, passing through large residential areas and workspace, which are effective measures provided by Beijing to support enterprises to resume work and production under the normal situation of epidemic prevention and control, the project requires that the location of the stops cannot be changed, that is, the location of the stops is fixed, however the service order of the stops can be optimized according to the passengers’ time window information. Meantime in order to compare the optimization results more intuitively and focus on the effectiveness of the algorithm, we directly adopt the location settings of the original stops. the stops’ information (numbered according to the order of service) of the pick-up and drop-off areas is shown in Table 1.

**TABLE 1:** Information of CB Stops

Route ID	Pick-up stop	Drop-off stop
1	Liuxing Garden(1), East Gate of Shangbei Center(2), Longteng yuan(3), New Longcheng(4)	Zhongguancun(5), Haidian Huangzhuang(6), Haidian Middle Street(7), West Exit of Haidian Avenue(8)
2	East Exit of Huashijiang community(1), Huashijiang Community(2), Tuqiao Subway Stop(3), Li yuan Subway Stop(4), Jiukeshu Subway Stop(5), Nikko Kiyoshi(6)	South Exit of Guangshun South Street(7), East Exit of Futong East Street(8), Guofeng Beijing(9), Fu’an Road(10), Wangjing Metro Stop(11), East Exit of Xiyang East Road(12), East Exit of Wangjing North Road(13)
3	Yizhung Jinmaoyue Community(1), Haizifu Community(2), Nanhai Community 2(3), Nanhai Community 3(4), Taihe Community(5), Zhongxin Garden(6), Taihe yuan Community(7)	Ciqikou Street(8), Chongwenmen(9)

Field data surveys are used to obtain the actual operating route of the CB, the time of departure from the stop and the time of arrival at the stop, the number of passengers who pick up and drop off bus. Investigators are assigned to the three routes. One sits at the front door and records the CB’s arrival time, the number of passengers who pick up bus, and the average boarding time, and the other sitting at the back door records the number of passengers who drop off bus, the average alighting time, and the departure time. The actual expected departure time of passengers is obtained by issuing questionnaires on the CB, and the time window is determined.

Route 1 contains 4 pick-up stops and 4 drop-off stops, Route 2 contains 6 pick-up stops and 7 drop-off stops, and Route 3 contains 7 pick-up stops and 2 drop-off stops. Table 2 lists the number of passengers and time window information for the three CB routes’ stops.

**TABLE 2:** Passenger Number and Time Windows of Three Routes

Route 1			Route 2			Route 3		
Stop ID	PN	TW	Stop ID	PN	TW	Stop ID	PN	TW
1	6	[7:05,7:15]	1	3	[7:00,7:10]	1	5	[7:00,7:10]
2	12	[7:15,7:25]	2	5	[7:15,7:25]	2	3	[7:00,7:10]
3	5	[7:10,7:20]	3	7	[7:00,7:10]	3	4	[7:10,7:20]
4	7	[7:10,7:20]	4	2	[7:20,7:30]	4	6	[7:05,7:15]
5	6	[7:45,7:55]	5	7	[7:20,7:30]	5	2	[7:10,7:20]
6	13	[8:10,8:20]	6	6	[7:25,7:35]	6	5	[7:00,7:10]
7	5	[7:50,8:00]	7	8	[8:00,8:10]	7	3	[7:15,7:25]
8	6	[8:15,8:25]	8	3	[7:55,8:05]	8	11	[7:45,7:55]
			9	6	[7:55,8:05]	9	17	[7:55,8:05]
			10	4	[8:05,8:15]			
			11	2	[8:10,8:20]			
			12	3	[8:10,8:20]			
			13	4	[8:15,8:25]			

NOTE: PN - Passenger Number; TW - Time Window

### B. Variable and Parameter Settings

**FIGURE 10.**
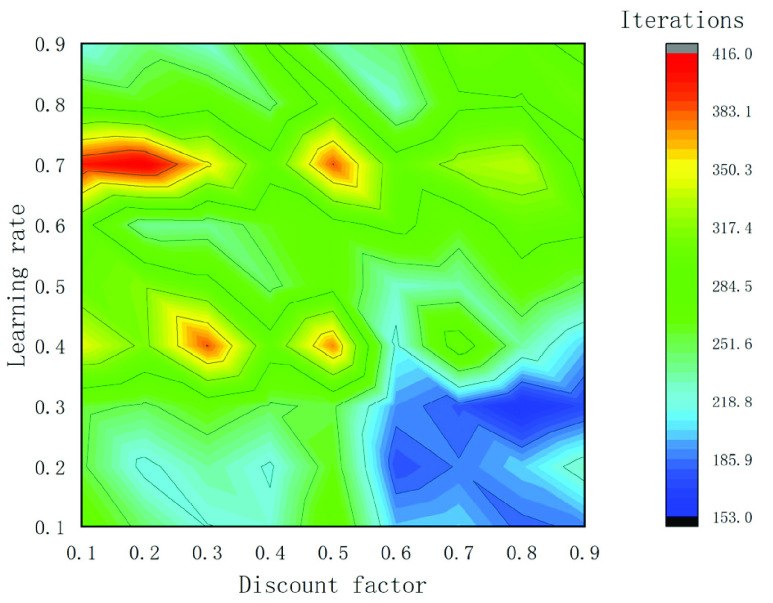
Parameter sensitivity analysis result.

A sensitivity analysis based on the condition with Route 1 is performed to explore the relation between model parameters and optimized results. The learning rate and discount factor are the most critical parameters in the Q-Learning algorithm. We adjust different parameter combinations in the Q-Learning algorithm to obtain the optimal parameter settings. The value range of the learning rate and discount factor is set as [0.1, 0.9], and perform training experiment according to the value of 0.1 unit. The parameter sensitivity analysis results are shown in Figure 10, the horizontal axis represents the discount factor, the vertical axis indicates the learning rate, and different colors represent different iterations, the following conclusions can be drawn that when the learning rate is within the range of [0.1,0.5] and the discount factor is within the range of [0.1,0.5], with the increase of the learning rate, the iterations on the whole presents an increasing trend; when the learning rate is within the range of [0.5,0.9] and the discount factor is within the range of [0.1,0.4], the iterations decrease as a whole with the increase of the discount factor; when the value range of the discount factor is [0.6,0.8] and the value range of the learning rate is [0.1,0.4], the iterations of the agent is relatively small compared to other parameter combinations, which indicates that the agent can complete the training faster, that is, the performance of the algorithm is better, and when the learning rate is 0.3, and the discount factor is 0.8, the algorithm efficiency is the highest. Besides, The CB speed and oil price are set according to the actual situation in Beijing [43], and the time value of passengers is

calculated according to Beijing’s per capita income in 2019. The parameter and variable settings are shown in Table 3.

**TABLE 3:** Variable and parameter settings

Variable	Description	Value
$v$	Bus free flow speed, set as a constant	30 km/h
$u_{p}^{+} $	Time value of passenger in pick-up area	1 yuan/min
$u_{p}^{-} $	Time value of passenger in drop-off area	2 yuan/min
$p$	Fuel consumption cost per unit operating mileage	3 yuan/km
Parameter	Description	Value
$\gamma $	Discount factor	0.8
$\alpha $	Learning rate	0.3
$Maxiter$	Max exploration steps	100
$Trials$	Maximum episodes	500
$\varepsilon $	Exploration factor	0.8
1	Constant, avoid the denominator being 0	0.01
$\omega _{1},\omega _{2} $	Weight coefficient	0.6, 0.4
$\varphi,\varpi $	parameter	0.15, 0.4

### C. Result Analysis

Based on actual data, the path with the least impedance is obtained by Eq (14), and Table 4 shows the distance between the stops.

**TABLE 4:** CB Stops’ Distance Matrix(km)

Stop ID of Route 1	1	2	3	4	5	6	7	8
1	0	1.1	1.8	4.3	–	–	–	–
2	1.1	0	1.2	2.9	–	–	–	–
3	1.8	1.2	0	2.1	–	–	–	–
4	4.3	2.9	2.1	0	–	–	–	–
5	–	–	–	–	0	0.5	1	0.5
6	–	–	–	–	0.5	0	0.5	0.8
7	–	–	–	–	1	0.5	0	0.6
8	–	–	–	–	0.5	0.8	0.6	0

Stop ID of Route 2	1	2	3	4	5	6	7	8	9	10	11	12	13
1	0	0.25	1.9	3.9	5.1	6.5	–	–	–	–	–	–	–
2	0.2	0	1.7	3.7	4.9	6.3	–	–	–	–	–	–	–
3	1.9	1.7	0	2.4	3.6	5.0	–	–	–	–	–	–	–
4	3.9	3.7	2.4	0	1.3	2.6	–	–	–	–	–	–	–
5	5.1	4.9	3.6	1.3	0	1.4	–	–	–	–	–	–	–
6	6.5	6.3	5.0	2.6	1.4	0	–	–	–	–	–	–	–
7	–	–	–	–	–	–	0	0.75	1.5	1.8	1.9	2.9	3.7
8	–	–	–	–	–	–	0.75	0	1.1	1.2	2.4	2.3	3.2
9	–	–	–	–	–	–	1.5	1.1	0	0.53	2.2	1.6	2.2
10	–	–	–	–	–	–	1.8	1.2	0.53	0	2.1	1.1	1.6
11	–	–	–	–	–	–	1.9	2.4	2.2	2.1	0	2.7	2.7
12	–	–	–	–	–	–	2.9	2.3	1.6	1.1	2.7	0	1.1
13	–	–	–	–	–	–	3.7	3.2	2.2	1.6	2.7	1.1	0

Stop ID of Route 3	1	2	3	4	5	6	7	8	9
1	0	0.44	1.2	1.7	2.1	3.2	2.8	–	–
2	0.44	0	1.1	1.3	2.4	2.8	2.6	–	–
3	1.2	1.1	0	0.56	1.6	2.1	1.8	–	–
4	1.7	1.3	0.56	0	0.46	0.4	1.1	–	–
5	2.1	2.4	1.6	0.46	0	0.5	1	–	–
6	3.2	2.8	2.1	0.4	0.5	0	0.5	–	–
7	2.8	2.6	1.8	1.1	1	0.5	0	–	–
8	–	–	–	–	–	–	–	0	2.0
9	–	–	–	–	–	–	–	2.0	0

**FIGURE 11.**
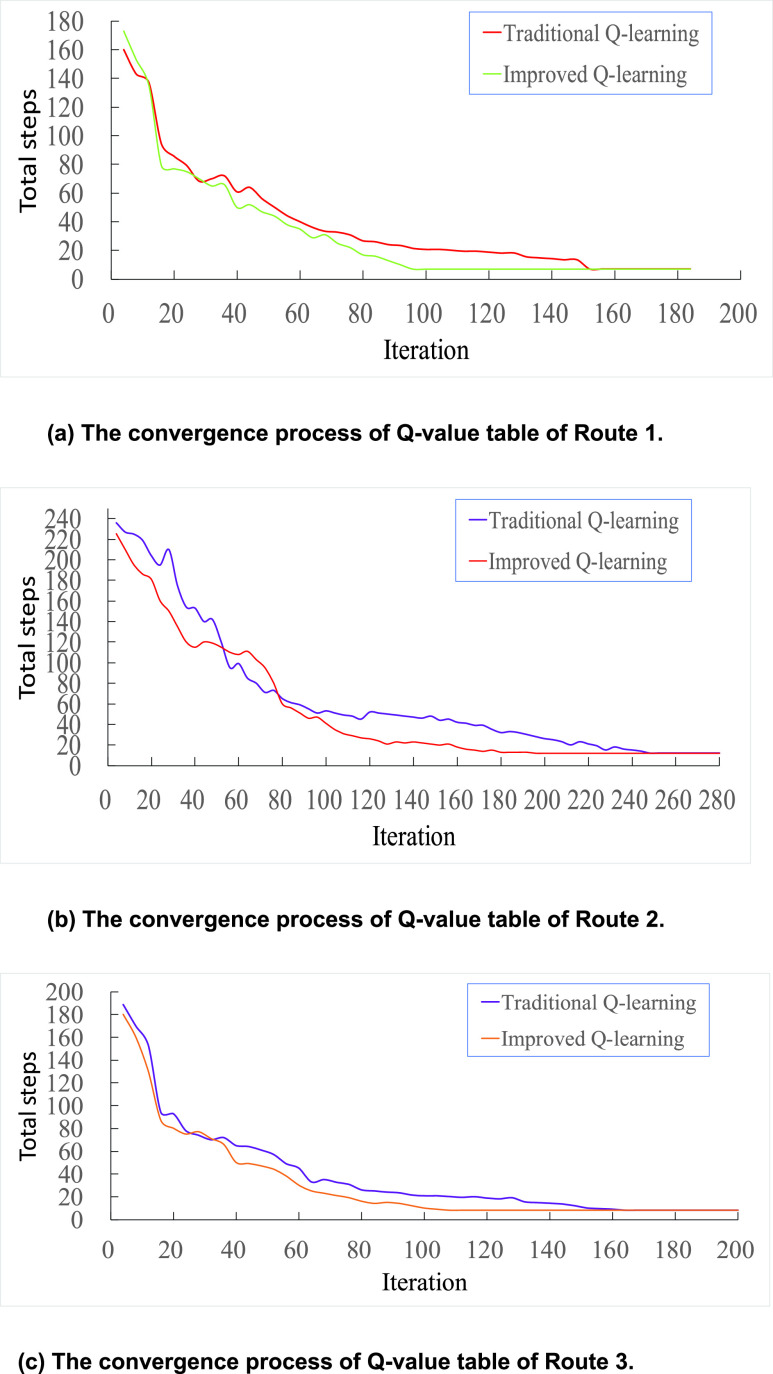
The convergence process of Q-value table.

This experiment is based on Python 3.6.4 programming, the computer configuration is Lenovo Y7000, the processor is i5-8300H, the graphics card NG GTX 1050, and the simulation environment we used is OpenAl Gym, the training results are shown in Figure (11 a-c) and Table 5.

Figure (11 a-c) and Table 5 show the convergence process of Q-value table and the comparison of algorithm performance. The total steps (y-axis) represents the number of steps which the agent needs to complete a training, iteration (x-axis) represents the number of training. From Figure (11 a-c), we can see that the number of steps for the agent to complete a training session continues to decrease with the number of iterations, until the number of exploration steps no longer changes. It directly means that the optimal strategy is learned by agent, namely, the Q-value table converges. When the Q value converges, the number of iterations of the improved Q-Learning algorithm is significantly less than that of the traditional Q-Learning algorithm. From Table 5 we can draw that compared with traditional Q-learning, the improved Q-learning training times of Route 1 is reduced by 37.9%, the solution time become shorter by 41.7%, and the optimization rate of training times and solving time of Route 2 has also reached more than 20%, the optimization rate of training times and solving time of Route 3 are both about 34%. Indicating that the improved Q-learning algorithm has improved, the problem of lag in data transmission significantly improves the convergence speed of the algorithm and shortens the route planning time.

**TABLE 5:** Comparison of Algorithm Performance

Route	Route 1		Route 2		Route 3	
Training metrics	Iterations	Solving time/s	Iterations	Solving time/s	Iterations	Solving time/s
Traditional Q-learning	153	187	246	266	162	196
Improved Q-learning	95	109	192	212	106	131
Optimization rate	37.9%	41.7%	22%	20.3%	34.6%	33.2%

The route solved by the improved Q-learning are as follows (stops’ number):

Route 1: 1-3-4-2-5-7-6-8;
Route 2: 1-3-2-5-4-6-8-9-7-10-11-12-13,
Route 3: 1-2-4-5-3-6-7-8-9,

the cost information after route optimization is shown in Table 6.

**TABLE 6:** Comparison of Route Cost Comparison

Route	Fuel consumption cost (yuan)	Time cost (yuan)	Total cost (yuan)	Total coat optimization rate
Current Route 1	86	149	235	39.1%
Optimized Route 1	92	51	143	
Current Route 2	147	244	391	35.9%
Optimized Route 2	174.8	76	250.8	
Current Route 3	134	164	298	28.2%
Optimized Route 3	125	89	208	

According to the results of the numerical experiment in Table 6, we can obtain the following observations. (i) Although the fuel consumption cost of the optimized Route 1 has increased by 6 *yuan* compared with the original route, the time cost of individual passenger has been reduced by 98 *yuan*, the average lost time of passengers has been reduced from 4.97 min to 1.7 min, and the total travel cost has been reduced by 39.1 %. (ii) The fuel consumption cost of the optimized Route 2 has increased by 27.8 *yuan* compared with the original route, but the time cost of passengers has been reduced by 168 *yuan*, the average lost time of individual passenger has been reduced from 5.42 min to 1.69 min, and the total travel cost has been reduced by 35.9%. (iii) The fuel consumption cost of the optimized Route 3 has increased by 9 *yuan* compared with the original route, but the time cost of individual passenger has been reduced by 75 *yuan*, the average lost time of passengers has been reduced from 5.85 min to 3.17 min, and the total travel cost has been reduced by 28.2%. This method can effectively reduce the total social travel cost. (IV) Compared with the original route, the waiting time of passengers on Route 1, Route 2 and Route 3 are reduced by 65.8%, 68.9% and 45.7% respectively. The average waiting time of the three routes is reduced by 60.1%. Assuming that the gathering time of passengers is positively correlated with the spread probability of COVID-19, it is believed that the probability of transmission of the COVID-19 is also correspondingly reduced sharply.

Through the above analysis, CB operation companies can consider increasing the fare moderately (after optimization, Route 1, Route 2 and Route 3 will increase the fare by 0.12 *yuan/person*, 0.58 *yuan/person* and 0.32 *yuan/person*, respectively) to make up for the business operating costs after route optimization. The increase, that is, maintaining the same operating cost as the status quo. In this case, because the bus fare is only a transfer payment and is not included in the total social travel cost, the reduction in the total social travel cost is all due to the contribution of the passenger travel cost reduction.

## VI. Discussions and Conclusion

In this study, we propose a CB route planning method based on the improved Q-learning algorithm to solve the problem of cross-regional CB route planning during the COVID-19. Under such a method, the total social travel cost, including the operation cost and travel cost, is minimized. First, we design a sub-regional route planning approach considering commuters’ time windows of pick-up stops and drop-off stops. Secondly, we develop the improved Q-learning algorithm, including state-action pair, reward function and update rule of Q value table. Specially, according to the operating cost of the bus company and the time cost of passengers, the reward function is set, and the “backtracking” idea is applied to the Q-learning algorithm to improve the update efficiency of the Q value table. Moreover, through the numerical experiment, we obtain the following conclusions. (i) The improved Q-learning algorithm can effectively increase the solution speed and improve the lag of data transmission. (ii) The optimized CB route can effectively reduce the total social travel cost and the transmission risk of COVID-19, thereby providing new ideas for CB route planning and pricing.

As the development of this study, in the future research, the passenger’s dynamic travel needs can be considered to make real-time planning of the CB route, besides, it is also possible to consider the situation of the passengers late or early to the stops and the different requirements for time window of passengers at the same stop.
